# Application of structural equation modelling to inform best management strategies for Marek’s disease in Amhara region, Ethiopia

**DOI:** 10.1038/s41598-023-37636-6

**Published:** 2023-06-30

**Authors:** Mastewal Birhan, Nega Berhane, Saddam Mohammed Ibrahim, Haileyesus Dejene, Bereket Dessalegn, Wubet Weledemedhin Woldemichael, Esayas Gelaye, Belayneh Getachew, Takele Abayneh, Molalegne Bitew

**Affiliations:** 1 College of Veterinary Medicine and Animal Sciences, University of Gondar, Gondar, Ethiopia; 2 Institute of Biotechnology, University of Gondar, Gondar, Ethiopia; 3 Department of Veterinary Epidemiology and Public Health, College of Veterinary Medicine and Animal Sciences, University of Gondar, Gondar, Ethiopia; 4 National Veterinary Institute, Bishoftu, Ethiopia; 5 Food and Agriculture Organization of the United Nations, Sub-Regional Office for Eastern Africa, P.O. Box: 5536, Addis Ababa, Ethiopia; 6 Bio and Emerging Technology Institute, Addis Ababa, Ethiopia

**Keywords:** Biotechnology, Computational biology and bioinformatics, Diseases, Risk factors

## Abstract

Marek’s disease, a highly contagious and an economically significant oncogenic and paralytic viral diseases of poultry, is becoming a serious problem in Ethiopia’s poultry sector. The aim of the study was to examine the relationship between risk factors and their contribution to develop risk with the intentions to implement MD control measures in the different chicken production systems of Ethiopia using the SEM framework. A questionnaire was designed based on the framework and each model constructed was measured using a set of rating scale items. Thus, a sample size of 200 farmers from different production systems were chosen for the data collection. From the analysis, Cornbrash’s Alpha (coefficient of reliability) based on the average inter-item correlations were evaluated for each parameter. The result showed that when litter management goes up by 1, the number of sick goes down by 37.575, the number of staff goes up by 1, the number of sick goes down by 7.63, litter management goes up by 1, the number of deaths goes down by 2.505, flock size goes up by 1, the number of deaths goes down by 0.007 than the rest of the activities. The result of this structural equation modeling finding indicates that the data fit the model well (χ^2^ = 0.201, RMSEA = 0.000, CFI = 1.00, TLI = 1.496, Degrees of freedom = 2) and the model was appropriated. In conclusion, flock size, litter management and number of staff activities have more impact on the numbers of sick, drops in egg production and the number of deaths. Therefore, practicing regular awareness creation for producers regarding management techniques is recommended.

## Introduction

Marek’s disease (MD) is a potential international hazard to poultry industry. It is characterized by the development of malignant neoplastic disease in visceral organs and nerves, resulting in paralysis and death in susceptible chickens^1^. Hence, to advance the management strategy of Marek’s diseases in poultry, several means would be required, such as increased use of currently available instruments, which include acceptable biosecurity measures, acceptable vaccination practices, and management of alternative immunological disorder viruses^2^. Vaccines have provided significant benefits to chicken industry in terms of the highest and best options to prevent Marek’s diseases^3^.

With the identification of Marek’s disease herpesvirus (MDV) in the late 1960s, the first vaccines were developed in England^4^ and by the end of the 1960s, the US Department of Agriculture (USDA) had established the fundamental purity and safety standards for poultry biologics^5^. Despite almost four decades of use, the specific mechanism(s) of MD vaccine-related immunity and anti-tumor benefits remain unknown^6^. With the exception of the cell-free lyophilized HVT, all vaccinations must be administered parentally in the form of living infected cells. Field virus exposure occurs early in life in the field, necessitating immunity in the young bird^7^. As a result, vaccination begins at one day old age. Vaccine protection against MD is complicated and affected by a number of factors. They include the host genotype, which can alter both the host’s susceptibility to MD and responsiveness to MD vaccine^8^.

Quantification of viral shedding patterns and virus-induced host mortality is required for a thorough understanding of disease epidemiology, not least to identify increases in virulence^9^. The disease is an airborne poultry infection that mostly affects chickens and costs the industry $1–2 billion annually^10^. Moreover, the chicken production is an important component of the livelihoods of most rural people (an estimated two-thirds of Ethiopian peasants keep poultry), and birds are regularly housed at night on perches within the family dwelling, often in the kitchen^11^. Ethiopia’s poultry industry is young but rapidly growing. The sector has a number of issues, including a lack of high-quality feed, inadequate husbandry techniques, and the presence and widespread dissemination of infectious and non-infectious diseases. Other obstacles are expected to be inadequate veterinary services and a lack of acceptable breeding methods and simultaneously the government also paid less attention in this low-profile sector. Different diseases cause high mortality rates ranging from 20 to 50%^12^. Inadequate disease/health management and limited resource stymie the development of chicken production by lowering productivity and increasing the likelihood of disease outbreaks^13^.

Furthermore, improved chicken output through the use of exotic breeds in Ethiopia failed to become a sustainable solution, owing to the problem of chicken not being extensively adopted by rural farmers due to a variety of socioeconomic and environmental obstacles. The management conditions under which the animals are produced differ along the existing production systems, which were roughly characterized as village, small-scale commercial, and large-scale commercial based on flock size, production objectives, and level of specialization and/or technology utilization^14^. Diseases such as Marek’s disease, which has frequent outbreaks and poses a serious threat and challenge to Ethiopia’s young growing poultry industry, are among the impediments to poultry development in Ethiopia. Therefore, the objectives of this study were, to explore the relationships between farm size, litter management and number of staff as a risk factor for their contribution to develop risk (egg production loss and chicken death) with the intentions to implement MD control measures in the different chicken production systems of Ethiopia using the SEM framework.

## Materials and methods

### Theoretical framework

The core principle behind SEM is that it is a powerful multivariate approach that is increasingly being used in scientific research to test and assess multivariate causal relationships. The key to perform a regression analysis and to determine how much of the change in the dependent variable can be explained by the independent variable or variables. Although multiple regression analysis is limited to variables that can be observed, the basic principles can be used to structural equation modeling^15^

**Figure 1:**
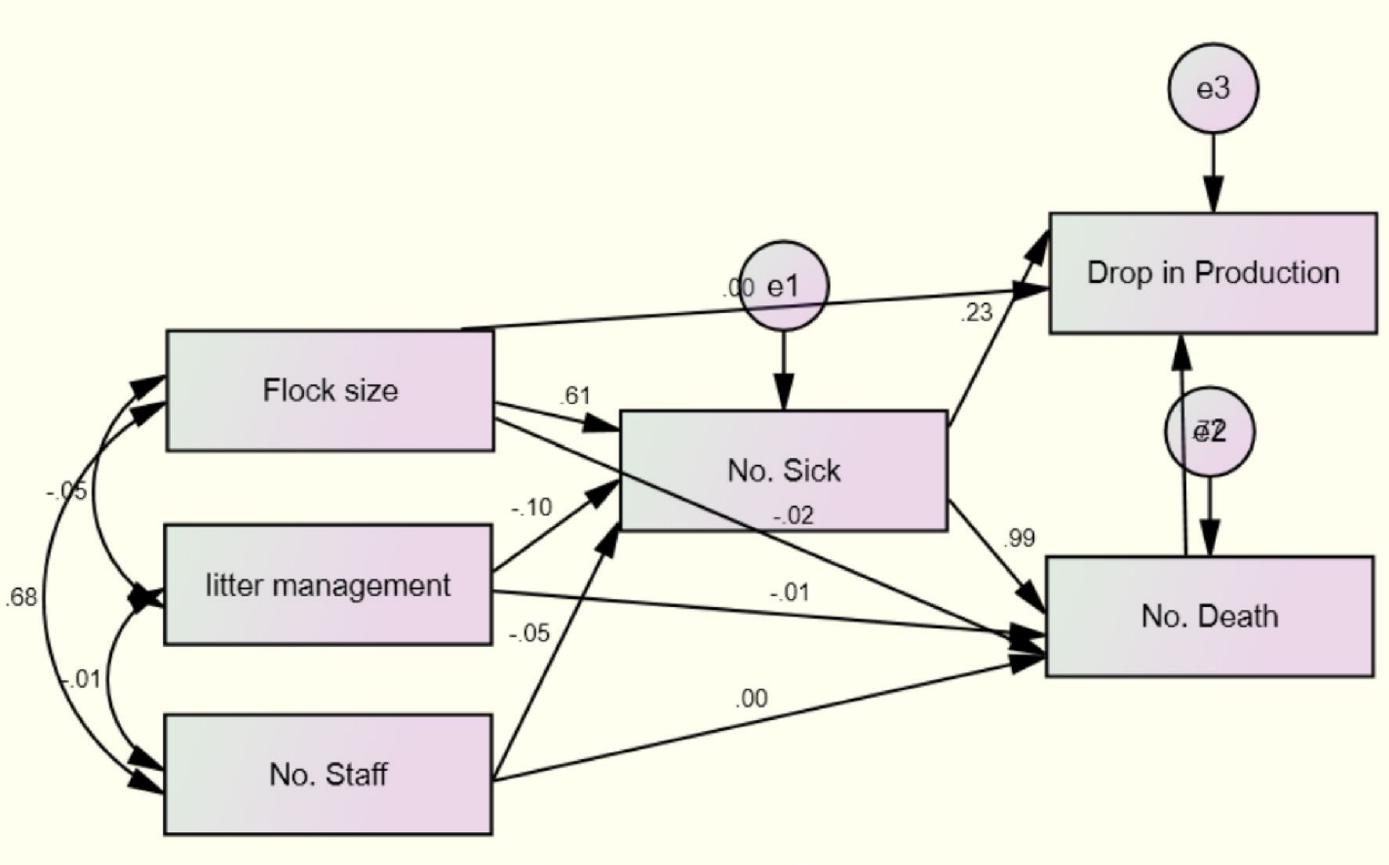
The constructs of SEM model in the performed factors analysis on the intention to implement MD control measures.

As a novel statistical analysis technique, it enables the testing of research hypotheses in a single procedure by modeling complex relationships between many observable and latent variables (Fig. 1). However, in the structural equation modeling method, both direct and indirect effects are combined. Based on theory, past empirical findings, or both, the researcher creates hypotheses regarding the relationships between variables. They are either direct or indirect connections in which intervening factors mediate the effect of one variable on another. Using past research and theoretical expectations as a reference, this researcher examined whether the interactions are unidirectional or bidirectional. The researcher develops the model by determining the amount and connections of measurable and latent variables^16^. 1. Assumed flock size about the likelihood of getting an MD disease, reduced egg production, or increased death rate; 2. Perceived number of staff in ability of the advised action to reduce MD threat, i.e., susceptibility and severity; 3. Patterns of correlation among a set of litter management of the recommended action for the control of MD; and 4. Confirms the correspondence of the data of the relations in the theoretical model.

Thinking of SEM as a combination of factor and path analysis prepares the researcher to consider SEM's two fundamental components: the measurement model and the structure model. SEM's measurement model enables researchers to assess how well their observed (measured) variables combine to identify underlying postulated components. The measurement model is tested using confirmatory factor analysis, and the hypothesized factors are relationships between flock size, litter management (LM), numbers of sick, drop-in egg production (DEP), and deaths.

As shown in Fig. 1, the first observed, endogenous variable (Number of sick, drop in egg production, Number of death) is linked to the observed, exogenous variables (flock size, litter management, number of staff), and unobserved, exogenous variables (e1, e2, and e3) (Question 1, Question 2, and Question 3) which are composed of the questions answered in the research questionnaire. The number of variables in this model is shown in Fig. 1: the number of observed variables (9), the number of unobserved variables (3), the number of exogenous variables (6), and the number of endogenous variables (3).

1 $$Question1= \lambda 1.X+e1$$

2 $$Question 2= \lambda 2.X+e2$$

3 $$Question 3= \lambda 3.X+e3$$

### Questionnaire design

A set of rating scale items was used to assess each model construct (questions). Three rating questions were used to extract the perceived flock size about the likelihood of contracting MD disease, reduced egg production, or increased death rate. The first question assessed the frequency of MD outbreaks in different flock sizes on different farms. The second question concerned the frequency of experience in the small flock size (< 50). Both questions were answered on a four-point scale, indicating the likelihood of an outbreak occurring per < 50, 51–1000, 1000–10,000, or > 10,001 chicken flock. The third question used a three-point grading scale (high, medium, and low) to determine the size of the chicken flock associated with MD occurrence. Two rating questions on a three-point scale were used to assess the perceived number of staff members or ability (large, medium, and small).

One of the questions concerned the influence of MD on general chicken egg production issues, while the other concerned the impact of MD relative to other poultry diseases. The patterns of correlation among a set of litter management of the advised action for the control of MD were measured by two rating scale questions on a three-point scale (high, medium, and low) for the proposed litter management control measures that influence chicken performance and are important for the welfare of the chickens. To confirm the empirical correlation of the relations in the theoretical model for MD control. The objectives for three suggested specific MD control approaches were weighed, including (1) farm-level chicken management, (2) MD vaccination address, and (3) biosecurity of chicken farms.

### Data collection

#### Sampling

For data gathering, a sample size of 200 farmers from various agricultural systems was chosen. The sample size was determined by a pragmatic assessment of SEM feasibility and test power for the specified statistical analysis. Furthermore, the minimum sample size for structural equation modeling is suggested to be 15^17^, while some scholars recommend that the sample size for SEM be 200–500, with at least 200^18^. Farmers in the intensive and extensive farming systems were sampled from 11 zones (North Shewa, North Gondar, East Gojjam, Central Gondar, Awi, West Gojjam, West Gondar, South Gondar, Bahir Dar, South Wollo, and North Wollo).

For data gathering, a sample size of 200 farmers from various agricultural systems was chosen. The sample size was determined by a pragmatic assessment of SEM feasibility and test power for the specified statistical analysis. Furthermore, the minimum sample size for structural equation modeling is suggested to be 15^17^, while some scholars recommend that the sample size for SEM be 200–500, with at least 200^18^. Farmers in the intensive and extensive farming systems were sampled from 11 zones (North Shewa, North Gondar, East Gojjam, Central Gondar, Awi, West Gojjam, West Gondar, South Gondar, Bahir Dar, South Wollo, and North Wollo). The researchers chose zones and cities based on their subjective assessment of the representativeness of the production systems and the ease of accessibility. The difference in the number of zones sampled from each production system reflected the proportion of zones in each production system. This method was repeated in other zones until the required sample size (200) for each sub-city was met.

The data collection instrument is prepared in English. The English version of the questionnaire is translated first to Amharic and back to English in order to ensure its consistency. The required data was collected from selected farmers and farm owners via face-to-face interview. Verbal informed consent was obtained from each farmer prior to the data collection. Verbal information and verbal informed consent is deemed appropriate due to the expectation of relatively low literacy levels among participants. The verbal informed consent and the entire study was approved by University of Gondar Institutional Review Board. In addition, all the methods were performed in accordance with the guidelines of the aforementioned institute.

### Model specification

The six fundamental constructs of the SEM were used in this research to assess farm owner perceptions of MD and its control in North West Ethiopia. In assessing the impact of these factors on farm owners' motivation to execute disease control measures via improving farm management, the intention to participate in hypothetical MD control measures was used as a surrogate for actual behavior. This is because there is no official oversight in place to directly measure behavior. Despite the fact that aim does not always explain farm management system was always tied to disease outbreaks. Socio-demographic and husbandry characteristics were employed as perception moderators in the study.

Furthermore, assumable from the preliminary study, the measurement model described the correlations between these six variables in Fig. 1. This paper proposed a model that can account for observed correlations between flock size, litter management (LM), number of staff, and the number of sick, drop in egg production (DEP), and death (both were indicators of their respective correlated factors). There was, however, a moderated relationship between all factors. The risk factor association was taken into consideration in our model. In essence, these have developed a model that states that the link between flock size, litter management (LM), and the staffing level, as well as the number of sick, drop in egg production (DEP), and deaths, is equal to 1.0 to scale latent variables. Our model determined the extent to which the hypothesized linkages captured the observed relationships on the parameter path^19^.

### Data analysis

The valuations of this model were to support adequate poultry health management to control the emergence of highly pathogenic diseases such as MD and to measure bio-security for better poultry production performance and quality in a competitive environment. Assessments of poultry production management skills to evaluate the proposed risk factor linkage to the incidence and reduction of poultry product due to MD. Measures were taken to reduce the risk factor for disease control in North West Ethiopia.

There are 13 correlation coefficients between the variables utilized in the SEM, which are the staff size, LM, FS, number of sick, number of deaths, DEP, and LM. Similarly, there is a correlation coefficient between the number of staff members, LM, FS, number of sick, number of deaths, DEP, and LM, with a commonly used significant value of 0.05. Each factor was then analyzed separately using a Factor Analysis (FA). Cronbach’s Alpha (coefficient of reliability) is calculated for each parameter based on the average inter-item correlations. Statistical Package for Social Science (SPSS) version 20 and Analysis of Moments Structures (AMOS) version 18 were used to analyze the confirmatory factor analyze, reliability test, descriptive statistics, Pearson Correlation, and path analysis.

### Ethical statement

This study was reviewed and approved by the Institutional Review Board of the University of Gondar, Ethiopia, with reference number, O/V/PRCS/05/495/2018.

## Results

### Variables summary

In the data collecting process, the observed variable (manifest variable) is the variable that is measured; latent variables are variables that are measured via connecting to observed variables because they cannot be assessed directly. Because latent variables represent abstract notions, they must be represented by more than one observable variable. In a research model, latent variables represent speculative notions^20^.

In this study, the observed or endogenous variables are the number of sick, the drop in egg production (DEP), and the number of deaths. Observed or exogenous variables include flock size (FS), litter management (LM), and staff number, whereas unobserved or exogenous variables include e1, e2, and e3. The variable counts are also as follows: number of variables in the model (9), number of observed variables (6), number of unobserved variables (3), number of exogenous variables (6), and number of endogenous variables (3). The number of distinct sample moments (21), the number of distinct parameters to be estimated (19), and the degrees of freedom (2).

To assess whether the variables in the data set are regularly distributed, the skewness and kurtosis values are checked. It is sufficient to mark the “test for normality” and “outliers” options in order to retrieve these test values in tabular form^21^. This is because tests like these are extremely sensitive to sample size, with larger sample sizes producing more significant results. As a result, it is suggested that significance tests be employed in conjunction with descriptive statistics, namely the kurtosis values for particular variables^22^. Kurtosis values less than 3.00 may imply that a variable has a normally distributed distribution^23^. As a result, the SEM result was normally distributed. Before analyzing the research model, the reliability of the new and changed components was thoroughly examined. In order to have a valid construct in the model, each of the items forming the model was checked to see if it was uni-dimensional, because this would have help to provide a consistent result.

### Normality test

The model examines the distribution of each observable variable for skewness and kurtosis to assess whether univariate normality exists. Skewness is the degree to which the distribution of a variable is asymmetrical, with positive skew indicating a distribution with many scores at the low end of a scale (the score distribution for a very difficult test). Absolute values greater than 3.0 for the skewness index are considered extreme^24^. Kurtosis is a measure of the distribution's peak and tails. Positive kurtosis indicates very peaked distributions (leptokurtic), with short, thick tails (also known as heavy-tailed distributions) indicating a small number of outliers. When the distribution is quite flat (mesokurtic), with long, thin tails indicating many outliers, negative kurtosis exists. Absolute values for the kurtosis index greater than 10.0 indicate a problem, and values greater than 20.0 are extreme^19^. Since distributions can deviate from normality in at least four ways, univariate normality is especially crucial to address. All variables in our model were skewed to the right (Table 1).

**Table 1:** Assessment of normality.

Variable	Min	Max	Skew	c.r	Kurtosis	c.r
No. staff	2	10	1.252	7.229	1.047	3.023
LM	1	3	0.812	4.685	− 0.602	− 1.737
FS	200	3000	1.222	7.056	1.401	4.044
No. Sick	10	1876	2.481	14.323	9.395	27.122
No. Death	2	1868	2.594	14.976	10.08	29.099
DEP	8	1874	2.607	15.053	10.178	29.382
Multivariate					259.285	187.123

### Sample moments

The covariance statistical result in Table 2 depicts and determines the relationship between the movement of observed and latent variable asset prices. When two variables move together, the covariance is positive; when they move inversely, the covariance is negative. As a result, except for the relationship between the number of staff and the LM, the relationships between the FS and the LM, the number of sick and the LM, the number of death and the LM, and the relationship between the DEP and the LM are all positive.

**Table 2:** Covariance’s result on observed and latent variable.

	No. staff	LM	FS	No. sick	No. death	DEP
No. staff	3.316					
LM	− 0.019	0.494				
FS	657.507	− 17.159	286,017			
No. Sick	168.391	− 23.468	79,575.2	66,581.68		
No. Death	162.648	− 24.132	76,424.3	64,863.972	65,356.038	
DEP	163.039	− 23.534	77,015.7	64,918.483	64,907.52	65,064.618

Correlation, on the other hand, is the coefficient that reflects the strength of a linear relationship between variables. To state that there is a relationship between variables, this coefficient must be statistically significant. The correlation coefficient ranges between − 1 and + 1^25^. All of the variables utilized in the SEM, including the number of staff, LM, FS, number of sick, number of deaths, DEP, and LM, have a positive relationship (+ 1).

As a result, the most crucial assumption of regression analysis, linearity, also applies to structural equation modeling. The model's sample correlation coefficient result revealed a linear relationship between the dependent and independent variables (Table 3). The effect of one variable on another variable without mediation is considered to as a direct effect. The indirect effects, on the other hand, results from the intervention of a variable that acts as a mediator between independent and dependent variables^26^.

**Table 3:** Correlations and mean result on dependent and in dependent variable of SEM.

	No. staff	LM	FS	No. sick	No. death	DEP	Mean
No. staff	1*						3.685
LM	− 0.015	1*					1.575
FS	0.675	− 0.046	1*				951.45
No. Sick	0.358	− 0.129	0.577	1*			268.51
No. Death	0.349	− 0.134	0.559	0.983	1*		253.82
DEP	0.351	− 0.131	0.565	0.986	0.995	1*	260.25

### Parameter estimation, model evaluation, and model comparison

#### Maximum likelihood estimates

The regression weight estimate result shows that when LM increases by one, the number of sick decreases by 37.575, when staff increases by one, the number of sick decreases by 7.63, when LM increases by one, the number of deaths decreases by 2.505, and when FS increases by one, the number of deaths decreases by 0.007 than the rest of the activities. The likelihood of obtaining a critical ratio of 7.811 in absolute value is less than 0.001. In other words, at the 0.001 level (two-tailed), the regression weight for FS in predicting the number of sick was significantly different from zero.

The model’s findings are provided in this section (Table 4). Regarding the variables, the negative relationship between FS and LM was estimated to be (− 17.159), and the inverse relationship between LM and number of staff was estimated to be (− 0.019); the other were positive effects to risk and disease-causing factors.

**Table 4:** Regression weights estimate and critical ratio of SEM on impact of risk.

	Path		Estimate	S.E	C.R	*P*
No. sick	<–	FS	0.294	0.038	7.811	***
No. sick	<–	LM	− 37.575	21.086	− 1.782	0.075
No. sick	<–	No. staff	− 7.63	11.026	− 0.692	0.489
No. death	<–	No. sick	0.98	0.016	62.216	***
No. death	<–	LM	− 2.505	4.724	− 0.53	0.596
No. death	<–	FS	− 0.007	0.01	− 0.763	0.445
No. death	<–	No. staff	0.701	2.454	0.286	0.775
DEP	<–	No. sick	0.224	0.034	6.606	***
DEP	<–	FS	0.001	0.004	0.398	0.691
DEP	<–	No. death	0.769	0.034	22.798	***

### Matrices factor score weights estimates

The result indicated in Table 5 showed the direct effect of an independent variable (exogenous) on a dependent variable (endogenous). The results revealed that the relationships between the number of deaths and the LM, FS, and number of personnel were not all statistically significant (*p* > 0.05). In addition, Table 6 showed, the claimed indirect effect of an independent variable (exogenous) on a dependent variable (endogenous). The results revealed that the relationships between the number of deaths and the LM, FS, and number of staffs were not all statistically significant (*p* > 0.05). The result also revealed that, when the number of staff and LM were increased by one the number of sick increased by (− 7.63 and − 37.575, individually), or the number of staff and LM were negatively related to the number of sick but the number of staff and LM were positively related to the FS (0.294). LM and FS were negatively related to the number of death (− 2.505 and − 0.007, respectively), but the number of staff was positively related to the number of death (0.701). The number of staff, LM, and FS were all positively correlated with DEP. When the number of staffs, LM, and FS were increased by one, the DEP increased significantly (***).

**Table 5:** Maximum likelihood estimates from the SEM relationship between variable.

	Path		Estimate	S.E	C.R	*P*
A) Covariances						
FS	<–>	LM	− 17.159	26.684	− 0.643	0.52
FS	<–>	No. staff	657.507	83.295	7.894	***
LM	<–>	No. staff	− 0.019	0.091	− 0.208	0.835

	Estimate	S.E	C.R	*P*		
B) Variances						
FS	286,016.508	28,673.424	9.975	***		
LM	0.494	0.05	9.975	***		
No. staff	3.316	0.332	9.975	***		
e1	43,628.99	4373.847	9.975	***		
e2	2155.264	216.067	9.975	***		
e3	488.983	49.021	9.975	***		

	Path		Estimate			
C) Correlations						
FS	<–>	LM	− 0.046			
FS	<–>	No. Staff	0.675			
LM	<–>	No. Staff	− 0.015			

*S.E* standard error, *C.R* critical ration, *P* *p*-value.

**Table 6:** Factor score weights estimates from the SEM between dependent and in dependent variable.

	No. staff	LM	FS	No. sick	No. death
A) Direct effects					
No. sick	− **7.63**	− **37.575**	**0.294**	***	***
No. death	**0.701**	− **2.505**	− 0.007*	**0.98**	***
DEP	***	***	0.001*	**0.224**	**0.769**
B) Indirect effects					
No. sick	***	***	***	***	***
No. death	− **7.48**	− **36.833**	**0.288**	***	***
DEP	− **6.923**	− **38.674**	**0.281**	**0.754**	***
C) Total effects					
No. sick	− **7.63**	− **37.575**	**0.294**	***	***
No. death	− **6.778**	− **39.338**	**0.28**	**0.98**	***
DEP	− **6.923**	− **38.674**	**0.283**	**0.978**	**0.769**

*Statistically significant (*p* < 0.05).

Significant values are in bold.

### Model fit summary

All of the risk factors, all of the variables used in the SEM, the number of staff, LM, FS, number of sick, number of deaths, DEP and LM cause the risk, and the data fits the model reasonably well (Table 7). Fit indices or fit statistics are the measurements used in structural equation modeling to analyze the models’ compliance with the data. In the literature, there are numerous fit indices. The definitions of the most commonly used fit indices are provided here. The size of the sample was taken into account in the structural equation modeling analyses. Because sample size influences several of the fit indices^27^.

**Table 7:** SEM of the model fit summary in all variable.

Model	DF	*P*	CMIN/DF	RMR	GFI	AGFI	PGFI	RMSEA
DM	2	0.904	0.101	0.171	1	0.996	0.095	
SM					1			0.786
IM	15		123.878	38,259.5	0.386	0.14	0.276	

*DM* default model, SM structural model, *IM* independent model, *DF* degrees of freedom, *GFI* goodness of fit index, *AGFI* adjusted goodness of fit index, *PGFI* parsimonious goodness of fit index, *RMSEA* root mean square error of approximation, *P* *p*-value.

## Discussion

Marek’s disease is a chicken disease caused by a herpesvirus that leads to a reduction in the immune response in acutely infected chickens, followed by the development of tumors in many of the infected birds. Very virulent strains (vvMDV+) have been reported in several countries, affecting broilers, breeders, and commercial layers. This disease severely reduces the production of both egg-laying and meat-producing birds, resulting in a significant economic impact on the poultry sector. Vaccination, combined with good farm cleaning and disinfection, proper reception practices, adequate downtime between flocks, an all-in-all-out policy, accurate vaccination programs adapted to the type of bird and field situation, good vaccine preparation and administration practices, and strict biosecurity measures, can greatly reduce the incidence of MD and thereby prevent economic losses caused by the disease. In places where monovalent immunization is ineffective, bivalent or polyvalent vaccine is indicated for effective virulent MD prophylaxis. Vaccine failures do occur, however, as field strains continue to develop toward virulent pathotypes. The ever-changing nature of MD has pushed us to create new vaccines or vaccine methods to combat the more virulent developing strains. However, the competition between vaccine research and the progression of MD is a huge threat to the poultry business^28^.

AMOS would have to provide this coefficient as well as a “critical value” that can be regarded as a significance test (a critical value of 1.96 corresponds to a *p*-value of 0.05). The data are normally distributed if Mardia’s coefficient is significant (i.e., the critical ratio is less than 1.96 in magnitude). In the current SEM, where the sample size is to be quite large, Mardia’s coefficient is virtually always assured to be significant. As a result, the significance test on its own provides very useful information. Thus, it is suggested that significance tests be employed in conjunction with descriptive statistics, namely the kurtosis values for particular variables^22^.

AMOS would have to provide this coefficient as well as a “critical value” that can be regarded as a significance test (a critical value of 1.96 corresponds to a *p*-value of 0.05). The data are normally distributed if Mardia's coefficient is significant (i.e., the critical ratio is less than 1.96 in magnitude). In the current SEM, where the sample size is to be quite large, Mardia’s coefficient is virtually always assured to be significant. As a result, the significance test on its own provides very useful information. Thus, it is suggested that significance tests be employed in conjunction with descriptive statistics, namely the kurtosis values for particular variables^22^. Kurtosis values less than 3.00 may imply that a variable has a normally distributed distribution^23^. As a result, the current SEM result was normally distributed.

Previously, acceptable fit criteria were a non-significant CFI more than 0.90^29^, RMSEA less than 0.10, and a maximum upper bound of the 90% confidence interval of 0.10. Although many statisticians (and presumably reviewers) still adhere to these rules (as indicated by the favorable evaluation of models with fit indices at or near these levels), readers should be aware that there remains dispute over acceptable fit among them. According to recent research by Hu and Bentler^30^, showed that the maximum threshold for RMSEA is 0.06. Despite this, the results of this SEM, LM, FS, and number of staff employed indicate that the data fits the model well (χ2 = 0.201, RMSEA = 0.000, CFI = 1.00, TLI = 1.496).

Cronbach’s Alpha (coefficient of reliability) is calculated for each parameter based on the average inter-item correlations. Except for FS, each item in each construct has an item-total correlation of ≥ 0.80. Also, the Cronbach Alpha score for FS was greater than 0.80. When FS increases by one, the number of sick increases by 0.294, when the number of sick increases by one, the number of deaths increases by 0.98, when the number of staff increases by one, the number of deaths increases by 0.701, when FS increases by one, the DEP increases by 0.001, and when the number of deaths increases by one, the DEP increases by 0.769.

## Conclusion

In the present study, the researchers reduced the risk of bias during sampling by taking in account of not taking from more than one farmer or producer in a single area. In conclusion, the FS model and the number of employees had a significant effect on the risk of the chicken farm industry (DEP, number of sick and number of deaths increased). This model is used to identify vital in observable or exogenous variables such as flock size (FS), litter management (LM), staff number, activities that have a greater impact on the number of sick, drops in egg production (DEP), and the number of deaths. In Ethiopia, questionnaires are used to assess various farms from various zones, such as construction, infrastructure, feeding condition, vaccine access, and the research and development model. Three different input parameters (flock size, litter management, and number of staff) were discovered from the literature that determine the impact of the number of sick, drops in egg production, and number of deaths utilized to evaluate the effects of different risk factors on farming activities. Following that, the association between each of the risk factors and the agricultural risks was investigated. As a result, it can be concluded that the model must pay attention to risk factors such as flock size, litter management, and staff number, which differs from the previous research result, and this could be due to the fact that the effects of environmental factors are more on procurement and integration than the other farm risk factors. As a result of the above conclusion, regular poultry producer awareness creation on-farm management techniques and MD prevention through vaccine by government bodies are recommended.

## Data Availability

All data generated or analysed during this study are available upon the request of the corresponding authors.

## Abbreviations

MD: Marek’s disease
MDV: Marek’s disease virus
SEM: Structural equation modelling
